# DNA Methylation and Histone Acetylation Patterns in Cultured Bovine Adipose Tissue-Derived Stem Cells (BADSCs)

**DOI:** 10.22074/cellj.2015.492

**Published:** 2015-01-13

**Authors:** Beheshteh Abouhamzeh, Mohammad Salehi, Ahmad Hosseini, Ali Reza Masteri-Farahani, Fatemeh Fadai, Mohammad Hasan Heidari, Mohsen Nourozian, Masoud Soleimani, Mohsen Khorashadizadeh, Majid Mossahebi-Mohammadi, Ardalan Mansouri

**Affiliations:** 1 Cellular and Molecular Biology Research Center, Shahid Beheshti University of Medical Sciences, Tehran, Iran; 2 Department of Cell Biology and Anatomical Sciences, Faculty of Medicine, Shahid Beheshti University of Medical Sciences, Tehran, Iran; 3 Department of Biotechnology, Faculty of Medicine, Shahid Beheshti University of Medical Sciences, Tehran, Iran; 4 Department of Hematology, Faculty of Medical Science, Tarbiat Modares University, Tehran, Iran; 5 Department of Medical Biotechnology, School of Advanced Medical Technology, Tehran University of Medical Sciences, Tehran, Iran; 6 Department of Stem Cell Biology, Stem Cell Technology Research Center, Tehran, Iran

**Keywords:** Somatic Cell Nuclear Transfer, Epigenetics, DNA Methyltransferases, Histone Deacetyltransferses

## Abstract

**Objective:**

Many studies have focused on the epigenetic characteristics of donor cells to improve somatic cell nuclear transfer (SCNT). We hypothesized that the epigenetic status and chromatin structure of undifferentiated bovine adipose tissue-derived stem cells (BADSCs) would not remain constant during different passages. The objective of this study was to determine the mRNA expression patterns of DNA methyltransferases (*DNMT1, DNMT3a, DNMT3b*) and histone deacetyltransferses (*HDAC1, HDAC2, HDAC3*) in BADSCs. In addition, we compared the measured levels of octamer binding protein-4 expression (*OCT4*) and acetylation of H3K9 (*H3K9ac*) in BADSCs cultures and different passages *in vitro*.

**Materials and Methods:**

In this experimental study, subcutaneous fat was obtained from adult cows immediately post-mortem. Relative level of *DNMTs* and *HDACs* was examined using quantitative real time polymerase chain reaction (q-PCR), and the level of OCT4 and *H3K9ac* was analyzed by flow cytometry at passages 3 (P3), 5 (P5) and 7 (P7).

**Results:**

The OCT4 protein level was similar at P3 and P5 but a significant decrease in its level was seen at P7. The highest and lowest levels of *H3K9ac* were observed at P5 and P7, respectively. At P5, the expression of *HDACs* and *DNMTs* was significantly decreased. In contrast, a remarkable increase in the expression of DNMTs was observed at P7.

**Conclusion:**

Our data demonstrated that the epigenetic status of BADSCs was variable during culture. The P5 cells showed the highest level of stemness and multipotency and the lowest level of chromatin compaction. Therefore, we suggest that P5 cells may be more efficient for SCNT compared with other passages.

## Introduction

It has been proved that selecting proper donor cells can positively increase the efficiency rate of somatic cell nuclear transfer (SCNT) derived embryos (1). One of the most considerable issues in SCNT is transmission of genetic information from the donor cells to oocytes, since the oocyte cytoplasm is not able to eliminate the epigenetic markers and restores the genetic material to the embryonic totipotent state (2, 3). It has been verified that the initial chromatin structure of the donor cell, which is influenced by epigenetic markers, has a critical role in cell reprogramming (4). There is some evidence that undifferentiated cells such as embryonic stem cells (ESCs) need less reprogramming due to their intrinsic remodeling ability. In addition, they have loose chromatin structure and an active transcription complex as well as totipotency capability (5, 6). It has also been reported that the rate of live births among the embryos derived from ESCs is 10 to 20 percent higher than nuclear transfer (NT) embryos derived from differentiated somatic cells like cumulus cells. However, this rate depends on the source of donor cell and the number of cell passages (7, 8). In SCNT the cells must go through multiple passages *in vitro*. It has been confirmed that long term cell culture could change the epigenetic status of the cells (9) and affect the efficiency of cloning (4).

The ethical considerations and technical constraints for attaining ESCs have led to a reduction in the use of embryonic stem cells in NT (10, 11). Finding a population of adult stem cells (ASCs) with similar properties with ESCs, could improve the efficiency of SCNT. Therefore, ASCs have become the focus of investigations as an alternative to ESCs. However, unlike the ESCs, ASCs have limited differentiation and self-renewal capacities. The most common type of ASC is mesenchymal stem cells (MSCs) (12). They are found in numerous tissues, particularly in bone marrow and adipose tissue. MSCs have an inherent ability to proliferate *in vitro*, and this trait (13) makes them a notable candidate donor cell for NT compared to the somatic cells that are being used at the current time.

Cell behavior is controlled by DNA sequences that are tuned through epigenetic regulation processes. Epigenetic regulations change gene expression but have no modifying effect on DNA sequence (14). DNA methylation and histone acetylation are among the most significant epigenetic modifications that alter chromatin structure. DNA methylation involves the addition of a methyl group to the 5ˊ position of the CpG islands region of a gene promoter mediated by DNA methyltransferases (*DMNTs*), and can decrease transcription factor binding and switch off the gene (15). Three different types of *DNMTs* such as *DNMT1, DNMT3a,* and *DNMT3b* have been recognized in mammals. *DNMT1* is responsible for maintaining methylation throughout cell division and recognizing hemimethylated DNA (16). *DNMT3a* in the same way as *DNMT3b* mainly acts in de novo methylation and brings about new DNA methylation during differentiation processes (17).

Histone acetylation takes place on lysine residues on the N terminal tails of histone proteins. Accordingly, acetylated histone neutralizes positively charged amino acids and also, reduces the affinity between DNA and histones and makes them detach. Histone acetyltransferases (HATs) are responsible for transferring acetyl groups to lysine residues. Unlike HATs, histone deacetylases (HDACs) remove these acetyl groups. One of the most well-known epigenetic factors is acetylation of histone H3 at Lysine 9 (H3K9ac) (18, 19). The level of H3K9acs in a promoter is highly associated with its transcriptional activation, and determines the pluripotency and reprogramming capability of ESCs (20). OCT4 is a transcription factor that presents in both human and murine MSCs and is considered as a marker for pluripotency and maintenance of self-renewal (21). OCT4 expression is critical for the performance of ESCs (20, 22, 23).

It has been reported that DNA methylation and histone acetylation are necessary for the function of a large number of ASCs (self-renewal and differentiation) that are being affected by environmental factors and organismal aging *in vivo*, but there is no comprehensive knowledge about the behavior of ASCs and epigenetic modifications during *in vitro* culturing (24).

Adipose tissue is an easily obtainable source of MSCs. However, the epigenetic modifications of bovine adipose derived stem cells (BADSCs) in culture have not been studied yet. Therefore, the aim of this study was to evaluate differences between the mRNA content of HDACs and *DMNTs* as well as the level of OCT4 and H3K9ac in three passages (3, 5, 7) of BADSCs.

## Materials and Methods

This experimental study has been approved by the Ethical Committee of Shahid Beheshti University of Medical sciences, Tehran, Iran. All the chemicals were obtained from Sigma chemical corporation (St. Louis, MO, USA) unless otherwise noted.

### Establishment of the primary cultures

Subcutaneous fat was collected from Holstein adult cows immediately post mortem at a local abattoir. The sample was then transferred for further examination to the Molecular and Cellular Biology Research Center of Shahid Beheshti University of Medical Sciences, Tehran, Iran. The tissue was dissected into 1-2 mm pieces and was washed twice in calcium and magnesium free Dulbecco’s phosphate-buffered saline (DPBS) containing 1% penicillin/streptomycin (P/S). The tissue pieces were digested by enzyme in high glucose Dulbecco’s modified Eagle medium (DMEM) containing 0.5% collagenase type II in 5% CO_2_ at 39˚C for 3 hours (to accord with bovine body temperature). DMEM with 10% fetal bovine serum (FBS) was added to inactivate the enzyme, and the cell suspension was centrifuged. The cells were re-suspended in DMEM supplemented with 10% FBS and 1% P/S, and were cultured in 25 cm^2^ flasks under 5% CO_2_ and 90% humidity at 39˚C. The cells were passaged when they reached 80-90% confluence. The culture medium was changed every 2 days. Cultures were passaged by trypsin and then counted and re-seeded at an initial concentration of 100,000 cells per 25 cm^2^ flask.

### Cell differentiation

The third passage of BADSCs was tested for the ability to differentiate into adipocytes and osteoblasts. Adipogenesis was induced by culturing the cells in DMEM supplemented with 5% FBS, 1% P/S, 250 nΜ dexamethasone, 0.5 mM isobutyl methylxanthine (IBMX), and 50 μM indomethacin (6). For inducing osteogenesis, the cells were cultured in DMEM with 5% FBS, 1% P/S, 10-7 M dexamethasone, 50 μg/ml L-ascorbic acid biphosphate and 10 mM beta-glycerophosphate (25). One flask was cultured in mere DMEM supplemented with 5% FBS and 1% P/S as the control group. After 21-day induction, differentiation was confirmed by histological staining. The cells were washed using DPBS (Ca^2+^ and Mg^2+^ free), and then fixed in 4% paraformaldehyde. After fixation, all the cells were washed four times with DPBS and stained by alizarin red and oil red for osteocyte and adipocyte identification, respectively (13, 26).

### Cell cryopreservation and thawing

BADSCs were frozen for further investigations. For freezing, the cells were detached by trypsin and resuspended in FBS supplemented with 10% dimethyl sulfoxide (DMSO). Approximately, 1,000,000 cells/ml were frozen inside each cryovial. The cells were thawed at 38˚C in a water bath and were washed in culture medium. After 6 days, the cells were cultured in DMEM with 0.5% FBS (starvation) for five days to synchronize them in the G0/G1 phase (27, 28).

### Quantitative real-time polymerase chain reaction (Q-PCR)

Total RNA was extracted from a pool of 1,000,000 cells from passages 3, 5, and 7 in presumptive G0/ G1 phase of the cell cycle using Qiazol (Qiagen, Germany), according to the manufacturer’s protocol. The first strand cDNA was synthesized using random hexamers (Vivantis, Malaysia) in a total reaction volume of 25 μl using M-MLV reverse transcriptase (Vivantis, Malaysia). The cDNA products were immediately used for RT-PCR or real-time PCR. Expression of the genes was evaluated using RT-PCR (data not shown), and the level of gene expression was investigated by real-time PCR.

QPCR reaction was performed to assess the expression of *DNMTs* (*DNMT1, DNMT3a*, and *DNMT3b*) and *HDACs* (*HDAC1, HDAC2*, and *HDAC3*) relative to *glyceraldehyde-3-phosphate dehydrogenase* (GAPDH). Primer sequences are shown in table 1. The cDNA was amplified in a reaction mix with a total volume of 15 μl containing 6.5 μl q-PCR master mix (amplicon III), 4.5 μl nuclease-free water, 2 μl cDNA and 1μl of each sense and antisense primer (20 pmol) for each gene. QPCR was performed by a Rotor-gene Q real time analyzer (Corbet, Australia). For all the genes, a three-step program was used as follows. Denaturation cycle: 15 minutes at 95˚C and for each 40 cycles of PCR: 20 seconds at 95˚C followed by 1 minute at 55˚C and 30 seconds at 72˚C. Each cDNA sample was examined in triplicate and the average cycle threshold was estimated and normalized by the *GAPDH* gene. Finally, melting curve analysis was performed by q-PCR analyzer. After the amplification process, the samples were electrophoresed on 2% agarose gel.

**Table 1:** Primers used in real-time RT-PCR


Gene	Primer sequence	Accession number

***GAPDH***	F: GTC GGA GTG AAC GGA TTC	NM_001034034.2
	R: TTC TCT GCC TTG ACT GTG C	
***HDAC1***	F: AGA GAA GAA AGA AGT CAC AGA AG	NM_001037444.2
	R: GGA TAA AGG TAG GGA TTT GG	
***HDAC2***	F: GGC GGT CGT AGA AAT GTG	NM_001075146.1
	R: TTC TGA TTT GGC TCC TTT G	
***HDAC3***	F: GAT GAC CAG AGT TAC AAG CAC	NM_001206243.1
	R: CCA GTA GAG GGA TAT TGA AGC	
***DNMT1***	F: CGG AAC TTC GTC TCC TTC	NM_182651.2
	R: CAC GCC GTA CTG ACC AG	
***DNMT3a***	F: TTA CAC AGA AGC ATA TCC AGG	NM_001206502.1
	R: GAG GCG GTA GAA CTC AAA G	
***DNMT3b***	F: ATC TTG TGT CGT GTG GGG	NM_181813.2
	R: CTC GGA GAA CTT GCC ATC	


GAPDH; Glyceraldehyde-3-phosphate dehydrogenase, HDAC; Histone deacetylases and DNMT; DNA methyltransferases.

### Flow cytometry

Flow cytometry was used for the investigation of H3K9 acetylation through intranuclear protein screening. The cells were fixed and immunolabelled by a protocol modified by Habib et al. (29). Briefly, cells at P3, 5 and 7 were detached using trypsin/ethylenediaminetetraacetic acid (EDTA). Then, they were washed twice using tween solution containing DPBS (Ca^2+^ and Mg^2+^ free) supplemented with 1% BSA and 0.1% Tween 20 to enhance the permeability. After that, the cells were fixed using 0.25% paraformaldehyde in DPBS at 37˚C for 10 minutes. The samples were maintained at 4˚C for 10 minutes, were added to 9 volumes of methanol/PBS (88% methanol/12% PBS vol/vol) and stored at 20˚C. Later on, the cells were washed twice with tween solution; the pellet was treated with 2N HCL for 30 minutes at 37˚C and neutralized with 0.1 M borate buffer (pH=8.5) for 5 minutes at room temperature. After centrifuging, the pellet was again washed twice with tween solution and incubated for 20 minutes at 37˚C by adding the blocking solution (tween solution supplemented with 10% newborn calf serum). Afterwards, the primary antibody (Rabbit polyclonal to histone H3 acetyl k9, Abcam, USA) was added to the cells for 30 minutes at room temperature, the cells were washed three times in DPBS and labeled with the secondary antibody (Goat polyclonal Secondary Antibody to Rabbit IgG, FITC, Abcam, USA) for 45 minutes at 37˚C. The cells were stained using sodium citrate solution (0.112%) containing propidium iodide (50 μg/ml) and RNase (10 μg/ml) for 30 minutes at room temperature. Finally, the pellets were washed and resuspended in DPBS containing 1% BSA to be prepared for the next step, i.e. flow cytometry. HeLa cells were used as a positive control.

A flow cytometry protocol (30) was used to assess intracellular proteins for the evaluation of *OCT4*. Cells at P3, P5 and P7 were trypsinized and washed with DPBS. The pellet was fixed in 1% paraformaldehyde at 4˚C for 30 minutes. Then, it was washed twice with DPBS and incubated with 2% Triton X-100/PBS at 4˚C for 10 minutes. After that, the primary antibody (Rabbit polyclonal to OCT4, Abcam, USA) was added to the cells for 60 minutes at 4˚C, and the cells were washed in PBS and labeled with the secondary antibody (Goat polyclonal Secondary Antibody to Rabbit IgG, FITC, Abcam, USA) for 45 minutes at 37˚C. Mouse embryonic stem cells were used as a positive control.

### Statistical analysis

Quantitative gene expression results were analyzed by REST 2009 software (Qiagen, Germany). In addition, *GAPDH* was used as internal control. P values<0.05 were considered as statistically significant. An attuned flow cytometer (Attune, applied biosystem, USA) with Flowjo software was used for analysis of flowcytometry. Statistical analysis was performed by Service Provisioning System Software 16 (SPSS16, Chicago, IL, USA). Mean ± SD values of OCT4 and H3K9ac were compared by analysis of variance (ANOVA) and Tukey HSD test. P values less than 0.05 were considered statistically significant.

## Results

In this study, multipotency potential of the BADSCs was confirmed by differentiation into osteogenic and adipogenic lineages. The expression of histone deacetyltransfrases (*HDAC1, HDAC2*, and *HDAC3*) and DNA methyltransferases (*DNMT1, DNMT3a,* and *DNMT3b*) was analyzed by q-PCR. The relative levels of *H3K9ac* and *OCT4* was determined by flow cytometry.

**Fig 1:**
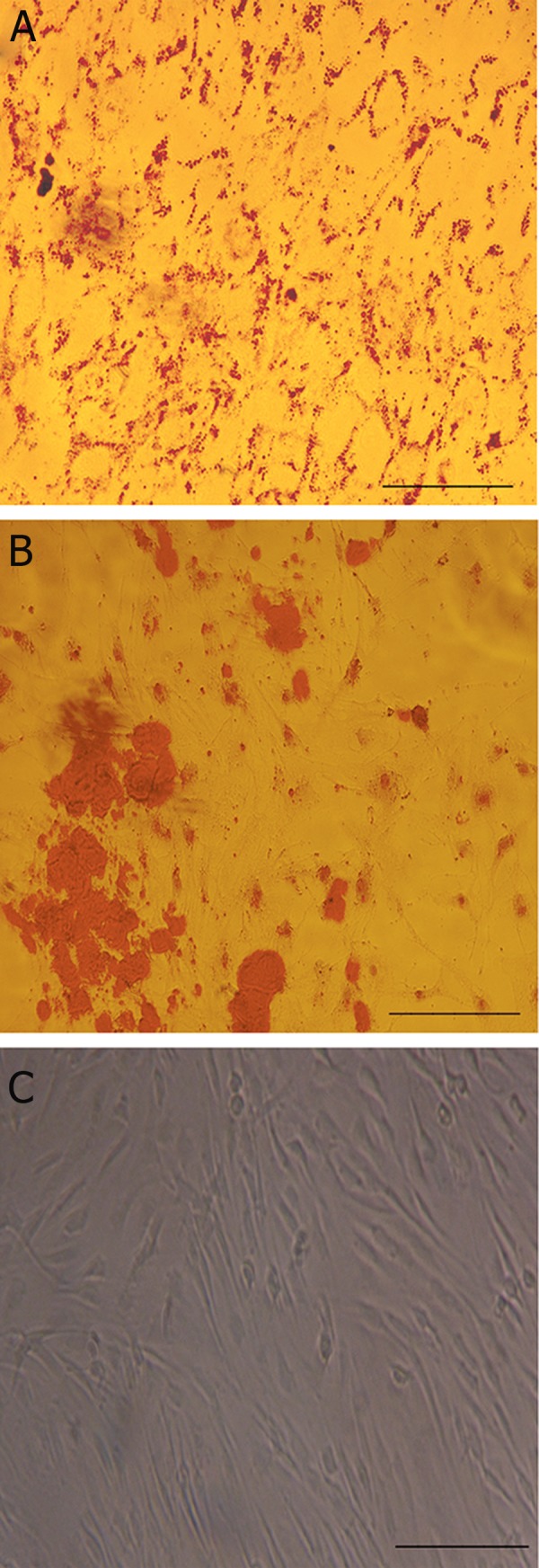
Microscopic images of BADSCs (A) differentiated into adipocytes stained by Oil Red (B) differentiated into osteocytes stained by Alizarin Red, and undifferentiated (C). Bar=50 μ. BADSCs; Bovine adipose tissue-derived stem cells.

Adipogenic potential was demonstrated with accumulation of fat droplets through oil-red staining (Fig 1A). Osteogenesis was confirmed by formation of calcium phosphate nodules (mineralized Ca^2+^ deposits) observed by alizarin red staining (Fig 1B). Figure1C showed the BADSCs without differentiation.

**Fig 2:**
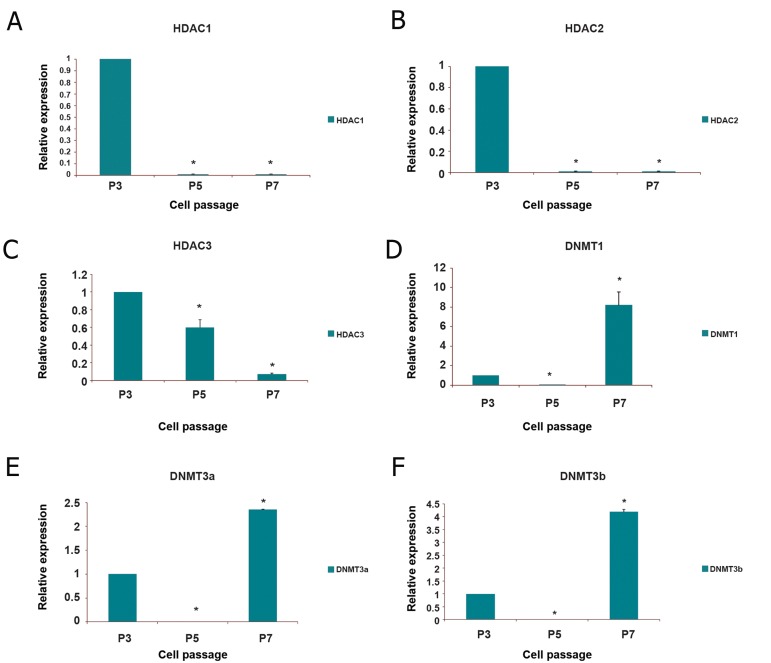
Histograms showing average relative transcription levels of HDAC1 (A), HDAC2 (B), HDAC3 (C), DNMT1 (D), DNMT3a (E) and DNMT3b (F) in BADSCs at P5 and P7 compared to P3. Gene transcription levels of the P3 cells were used as the calibrator. P; Passage number, HDAC; Histone deacetylases, DNMT; DNA methyltransferases and BADSCs; Bovine adipose derived stem cells.

The mRNA level of *DNMTs* and *HDACs* at P5 and P7 were compared to P3. Transcript level of *HDAC1* and *HDAC2* were significantly decreased (nearly 100-fold) at P5 and P7 compared to P3 (p<0.05) (Fig 2A, B).The expression level of *HDAC3* showed an approximately 1.6-fold decrease at P5, and was decreased about 14-fold at P7 (p<0.05) (Fig 2C). Our data indicated that at both P5 and P7, *HDAC1* and *HDAC2* had minimum and *HDAC3* had maximum levels of expression among *HDACs*, respectively. Moreover, the cells at P5 indicated about a 100-fold decrease in expression levels of DNMT1, DNMT3b and a 50- fold decrease in expression of DNMT3a compared to P3 (p<0.05) (Fig 2D-F). Thus, DNMT1 and DNMT3b showed identical expression levels at P5 while DNMT3a expression was two folds higher than both of them (p<0.05). The mRNA level of DNMT1, DNMT3a and DNMT3b at P7 was significantly increased, i.e.8, 2.3 and 4 fold compared to P3, respectively (p<0.05) (Fig 2D-F). Thus, the level of DNMT1 was about 2 fold and 3.47 fold higher than the level of DNMT3b and DNMT3a at P7, respectively (p<0.05).

**Fig 3:**
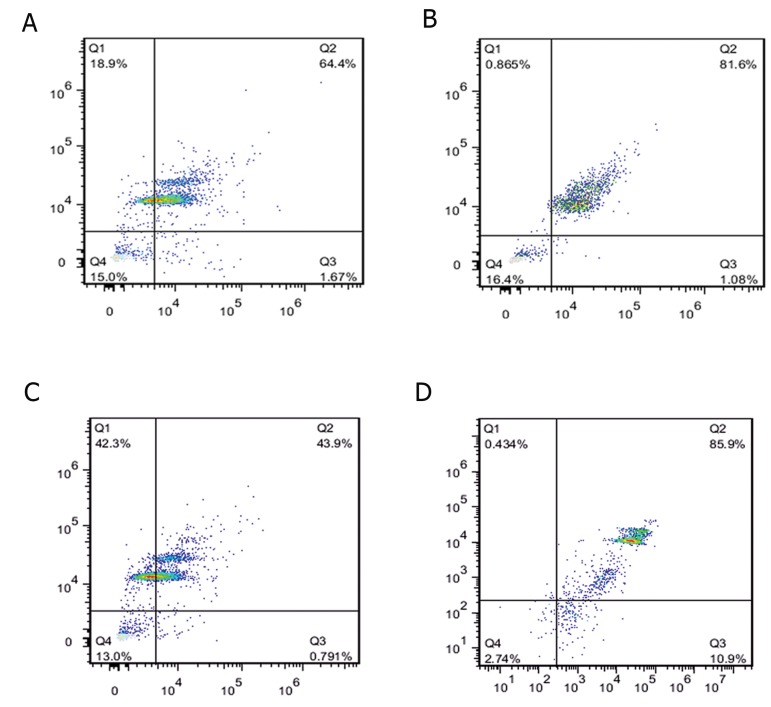
Histogram indicating distribution of acetylation H3K9 using flow cytometry in BADSCs at P3 (A), P5 (B), P7 (C) and (D) positive control (HeLa cell). P; Passage number, H3K9; Histone H3 at Lysine 9 and BADSCs; Bovine adipose derived stem cells.

**Fig 4:**
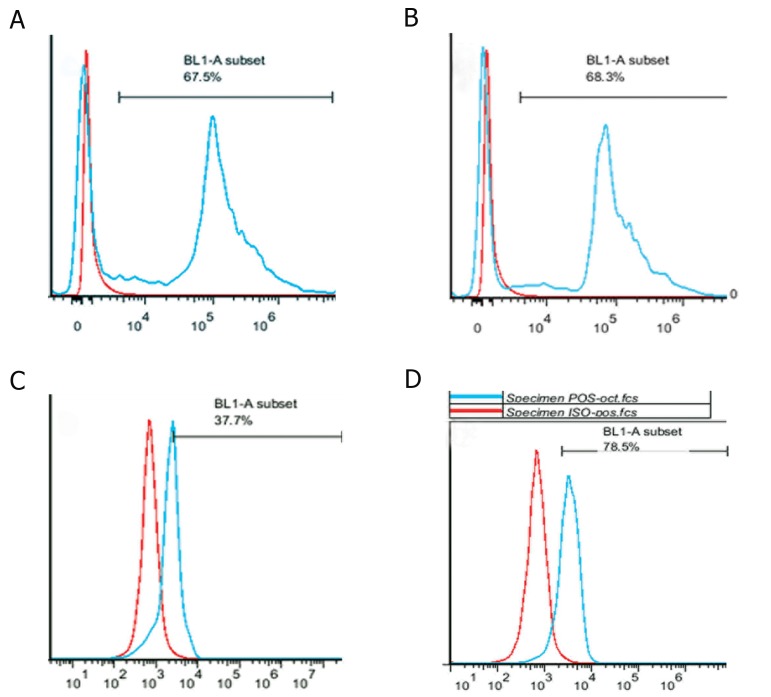
Histogram indicating distribution of Oct4 using flow cytometry in BADSCs at P3 (A), P5 (B), P7 (C) and (D) positive control (mouse embryonic stem cell). P; Passage number and BADSCs; Bovine adipose derived stem cells.

Acetylation of histone H3 on K9 and *OCT4* was variable in the cells at P3, P5, and P7. The acetylation rate of H3K9 was significantly higher at P5 (79.85% ± 2.50) compared to P3 (62.65% ± 2.47) and P7 (46.85% ± 4.17) (p<0.05, Fig 3A-C). The acetylation rate of *H3K9* in HeLa cells as positive control was 85.9% (Fig 3D). Analyzing the levels of *OCT4* showed no significant difference between P3 (63.05% ± 3.18) and P5 (65.15% ± 3.32) (p>0.05) but showed a dramatic decrease at P7 (39.1% ± 1.97) (p<0.05, Fig 4A-C).The expression of OCT4 in mouse ES cells as positive control was 78.5% (Fig 4D).

## Discussion

*In vitro* cultures influence the expression mechanisms of chromatin remodeling proteins as well as stemness and pluripotency of BADSCs (31-34). In comparison with *in vivo*, it has been revealed that *in vitro* culture of somatic cells changes the gene expression and DNA condensation patterns. Expression of chromatin remodeling proteins changes during different passages of somatic cells (as donor cells) *in vitro* which leads to different developmental courses for NT embryos (4, 34). In the present study, we evaluated the gene expression patterns of enzymes that are responsible for regulating epigenetic modification and subsequent chromatin changes in BADSs at different passages.

Evaluation of three different passages of BADSCs revealed that the expression of chromatin remodeling proteins and the level of *H3K9ac* and *OCT4* was changed in cell cultures in a time-dependent manner. The highest and lowest levels of *OCT4* and *H3K9ac* were observed at P5 and P7, respectively. In addition, the mRNA levels of *HDACs* and *DNMTs* were relatively high in the cells at P3 but lower at P5 than P3. P7 showed an increase in *DNMTs* and a constant level of *HDACs* except *HDAC3*.

Few investigations have assessed the level of histone acetylation and DNA methylation of cells in the cell cultures. Giraldo et al. (4) showed that the mRNA level of *DNMT1, MeCP2* and *HDAC1* is changed in fetal bovine fibroblast cells during the proliferative stages, before cellular senescence, however, no significant changes were detected in the methylated DNA or acetylated histone within the examined population. Enright et al. (35) reported that the level of acetylated histones in long-term cultured bovine fibroblast cells and cumulus cells was significantly higher than shortterm cultured cells (P15 vs. P5). Other researchers have indicated that the level of DNA methylation in normal murine, hamster and human cell lines was increased in culture over time (9, 36). It is likely that the procedures and times of cell trypsinization can affect chromatin reorganization in addition to the duration of culture and lead to changes in nuclear and cytoplasmic proteins (32, 33). The high mRNA level of *DNMTs* and *HDACs* at P3 cells might be due to the primary stress of culture establishment. However, the cells returned to their normal cellular processes after two or three passages at P5.

It has been verified that acetylation and methylation of histone H3 at lysine (K4, K9, K27) is changed during long-term culture of ADSCs, and H3 modification differs among the adipogenic cells differentiated from early or late passages of ADSCs (34, 37). In the same study, it was proposed that the histone modification occurring in late passages of MSCs might be responsible for decreasing their differentiation capacity (34, 37). Our research indicated that the level of H3K9 acetylation was not constant in cultured BADSCs. Reduction of *H3K9* acetylation at P7 could be due to reduced pluripotency potential of the stem cells and commitment to a particular lineage associated with low expression of *OCT4*. Increase in expression level of *DNMTs (DNMT1, DNMT3a, DNMT3b)* in P7 cells demonstrated that *de-novo* methylation occurs during late passage of adult stem cells, and is then maintained by *DNMT1* (as results showed that the level of *DNMT1* at P7 was higher than *DNMT3a* and *DNMT3b*). This DNA methylation might be the early beginning of a cascade leading to transcriptional silencing, mediated by targeting methyl-CpG-binding proteins (MeCPs) bound to methylated CpG sites in the promoter regions serving *HDACs*, subsequent to which the chromatin is condensed and the gene is silenced (38, 39). In addition, specific genes are turned on and the stem cells are probably committed to a specific lineage (40, 41). Another possibility for the epigenetic alterations at P7 could be replicative senescence. One of the characteristics of stem cells is a self-renewal feature, which is vital for their function. Self-renewal is defined as an asymmetrical division of an adult stem cell giving rise to a new stem cell and a daughter cell with less self-renewal capacity. However, symmetrical division of stem cells in culture dishes causes a rapid increase in the stem cell population. These symmetrical divisions can lead to stemness loss and cellular aging. Hayflick and Moorhead (42) have reported that human cultured primary cells are able to survive only for a limited number of passages before the death of the cells. Williams et al. (13) has demonstrated that modification of DNA methylation and histone H3 acetylation occur in late passages in porcine ASCs as they approach senescence. They demonstrated that porcine ADSCs reached cellular senescence at P9 while other studies indicated that DNA methylation in ADSCs remained constant up to at least 4 passages *in vitro* (43). Our results indicated that BADSCs at P7 or higher passages are committed to a differentiation pathway or tended to cellular senescence. BADSCs at P5 have the highest level of stemness and pluripotency and lower levels of gene expression patterns than chromatin remodeling proteins, and could be more efficient compared to other passages for NT. Decreased acetylation of *H3K9* and increased mRNA level of *DNMT1* at P7 may lead to reduced development of NT embryos. Fusion of cells at P7 as donor cell with a recipientooplasm introduces the somatic form of *DNMT1*, which could maintain the somatic methylation patterns in early NT embryos and lead to aberrant methylation and imprinting, ultimately disturbing NT embryos' development. However, further studies are required to completely elucidate the effects of passage number on BADSCs in relation to the outcome of NT. Future research could also examine the differentiation status of BADSCs at different passages.

## Conclusion

Our results demonstrated that the mRNA content of chromatin remodeling proteins and level of *OCT4* and *H3K9ac* are not constant in adult stem cells during culture and are changed by cell passage. These changes are likely to affect the competence of adult stem cells used as donor karyoplasm in NT.

## References

[1] Hill JR (2002). Abnormal in utero development of cloned animals: implications for human cloning. Differentiation.

[2] Cezar GG (2003). Epigenetic reprogramming of cloned animals. Cloning Stem Cells.

[3] Dean W, Santos F, Stojkovic M, Zakhartchenko V, Walter Jr, Wolf E (2001). Conservation of methylation reprogramming in mammalian development: aberrant reprogramming in cloned embryos. Proc Natl Acad Sci USA.

[4] Giraldo AM, Lynn JW, Purpera MN, Godke RA, Bondioli KR (2007). DNA methylation and histone acetylation patterns in cultured bovine fibroblasts for nuclear transfer. Mol Reprod Dev.

[5] Martin GR (1981). Isolation of a pluripotent cell line from early mouse embryos cultured in medium conditioned by teratocarcinoma stem cells. Proc Natl Acad Sci USA.

[6] Schwab KE, Hutchinson P, Gargett CE (2008). Identification of surface markers for prospective isolation of human endometrial stromal colony-forming cells. Hum Reprod.

[7] Hochedlinger K, Jaenisch R (2002). Monoclonal mice generated by nuclear transfer from mature B and T donor cells. Nature.

[8] Saito S, Sawai K, Ugai H, Moriyasu S, Minamihashi A, Yamamoto Y (2003). Generation of cloned calves and transgenic chimeric embryos from bovine embryonic stem-like cells. Biochem Biophys Res Commun.

[9] Wilson VL, Jones PA (1983). DNA methylation decreases in aging but not in immortal cells. Science.

[10] Frankel MS (2000). In search of stem cell policy. Science.

[11] Teven CM, Liu X, Hu N, Tang N, Kim SH, Huang E (2011). Epigenetic regulation of mesenchymal stem cells: a focus on osteogenic and adipogenic differentiation. Stem Cells Int.

[12] Luu HH, Song WX, Luo X, Manning D, Luo J, Deng ZL (2007). Distinct roles of bone morphogenetic proteins in osteogenic differentiation of mesenchymal stem cells. J Orthop Res.

[13] Williams KJ, Picou AA, Kish SL, Giraldo AM, Godke RA, Bondioli KR (2008). Isolation and characterization of porcine adipose tissuederived adult stem cells. Cells Tissues Organs.

[14] Riggs AD, Martienssen RA, Russo VEA, Russo VEA, Martienssen RA, Riggs AD (1996). Epigenetic mechanisms of gene regulation. Epigenetic mechanisems.

[15] Hiendleder S, Mund C, Reichenbach H-D, Wenigerkind H, Brem G, Zakhartchenko V (2004). Tissue-specific elevated genomic cytosine methylation levels are associated with an overgrowth phenotype of bovine fetuses derived by in vitro techniques. Biol Reprod.

[16] Chung YG, Ratnam S, Chaillet JR, Latham KE (2003). Abnormal regulation of DNA methyltransferase expression in cloned mouse embryos. Biol Reprod.

[17] Okano M, Bell DW, Haber DA, Li E (1999). DNA methyltransferases Dnmt3a and Dnmt3b are essential for de novo methylation and mammalian development. Cell.

[18] Heintzman ND, Stuart RK, Hon G, Fu Y, Ching CW, Hawkins RD (2007). Distinct and predictive chromatin signatures of transcriptional promoters and enhancers in the human genome. Nat Genet.

[19] Nishida H, Suzuki T, Kondo S, Miura H, Fujimura Y, Hayashizaki Y (2006). Histone H3 acetylated at lysine 9 in promoter is associated with low nucleosome density in the vicinity of transcription start site in human cell. Chromosome Res.

[20] Chambers I, Colby D, Robertson M, Nichols J, Lee S, Tweedie S (2003). Functional expression cloning of Nanog, a pluripotency sustaining factor in embryonic stem cells. Cell.

[21] Lengner CJ, Camargo FD, Hochedlinger K, Welstead GG, Zaidi S, Gokhale S (2007). Oct4 expression Is not required for mouse somatic stem cell self-renewal. Cell Stem Cell.

[22] Mitsui K, Tokuzawa Y, Itoh H, Segawa K, Murakami M, Takahashi K (2003). The homeoprotein Nanog is required for maintenance of pluripotency in mouse epiblast and ES cells. Cell.

[23] Nayernia K, Lee JH, Drusenheimer N, Nolte J, Wulf G, Dressel R (2006). Derivation of male germ cells from bone marrow stem cells. Lab Invest.

[24] Pollina EA, Brunet A (2011). Epigenetic regulation of aging stem cells. Oncogene.

[25] Mobarakeh ZT, Ai J, Yazdani F, Sorkhabadi SM, Ghanbari Z, Javidan AN (2012). Human endometrial stem cells as a new source for programming to neural cells. Cell Biol Int Rep (2010).

[26] Vidal MA, Kilroy GE, Lopez MJ, Johnson JR, Moore RM, Gimble JM (2007). Characterization of equine adipose tissue-derived stromal cells: adipogenic and osteogenic capacity and comparison with bone marrow-derived mesenchymal stromal cells. Vet Surg.

[27] Boquest AC, Day BN, Prather RS (1999). Flow cytometric cell cycle analysis of cultured porcine fetal fibroblast cells. Biol Reprod.

[28] Tian XC, Kubota C, Enright B, Yang X (2003). Cloning animals by somatic cell nuclear transfer--biological factors. Reprod Biol Endocrinol.

[29] Habib M, Fares F, Bourgeois CA, Bella C, Bernardino J, Hernandez- Blazquez F (1999). DNA global hypomethylation in EBV-transformed interphase nuclei. Exp Cell Res.

[30] Mossman AK, Sourris K, Ng E, Stanley EG, Elefanty AG (2005). Mixl1 and oct4 proteins are transiently co-expressed in differentiating mouse and human embryonic stem cells. Stem Cells Dev.

[31] Iwatani M, Ikegami K, Kremenska Y, Hattori N, Tanaka S, Yagi S (2006). Dimethyl sulfoxide has an impact on epigenetic profile in mouse embryoid body. Stem Cells.

[32] Maizel A, Nicolini C, Baserga R (1975). Effect of cell trypsinization on nuclear proteins of WI-38 fibroblasts in culture. J Cell Physiol.

[33] Chu EH (1962). Chromosomal stabilization of cell strains. Natl Cancer Inst Monogr.

[34] Giraldo AM, Hylan DA, Ballard CB, Purpera MN, Vaught TD, Lynn JW (2008). Effect of epigenetic modifications of donor somatic cells on the subsequent chromatin remodeling of cloned bovine embryos. Biol Reprod.

[35] Enright BP, Jeong BS, Yang X, Tian XC (2003). Epigenetic characteristics of bovine donor cells for nuclear transfer: levels of histone acetylation. Biol Reprod.

[36] Mazin AL (1995). Life span prediction from the rate of age-related DNA demethylation in normal and cancer cell lines. Exp Gerontol.

[37] Noer A, Lindeman LC, Collas P (2009). Histone H3 modifications associated with differentiation and long-term culture of mesenchymal adipose stem cells. Stem Cells Dev.

[38] Nan X, Ng HH, Johnson CA, Laherty CD, Turner BM, Eisenman RN (1998). Transcriptional repression by the methyl-CpG-binding protein MeCP2 involves a histone deacetylase complex. Nature.

[39] Ng HH, Zhang Y, Hendrich B, Johnson CA, Turner BM, Erdjument- Bromage H (1999). MBD2 is a transcriptional repressor belonging to the MeCP1 histone deacetylase complex. Nat Genet.

[40] Li E (2002). Chromatin modification and epigenetic reprogramming in mammalian development. Nat Rev Genet.

[41] Williams RR, Azuara V, Perry P, Sauer S, Dvorkina M, Jorgensen H (2006). Neural induction promotes large-scale chromatin reorganisation of the Mash1 locus. J Cell Sci.

[42] Hayflick L, Moorhead PS (1961). The serial cultivation of human diploid cell strains. Exp Cell Res.

[43] Noer A, Sorensen AL, Boquest AC, Collas P (2006). Stable CpG hypomethylation of adipogenic promoters in freshly isolated, cultured, and differentiated mesenchymal stem cells from adipose tissue. Mol Biol Cell.

